# Delayed Imaging Response After Chemoradiotherapy in a Patient with Unresectable Brainstem Pilocytic Astrocytoma

**DOI:** 10.3390/diagnostics16183011

**Published:** 2026-09-17

**Authors:** Yan Shen, Chao Li, Fengbo Huang, Wei Qian, Bicheng Zhang, Ting Zhang, Dang Wu

**Affiliations:** 1 Department of Radiation Oncology, Second Affiliated Hospital, School of Medicine, Zhejiang University, Hangzhou 310009, China; 2 Key Laboratory of Cancer Prevention and Intervention, National Ministry of Education, Cancer Institute, Second Affiliated Hospital, Zhejiang University School of Medicine, Zhejiang University, Hangzhou 310009, China; 3 Department of Pathology, Second Affiliated Hospital, Zhejiang University School of Medicine, Zhejiang University, Hangzhou 310009, China; 4 Department of Radiology, Second Affiliated Hospital, Zhejiang University School of Medicine, Zhejiang University, Hangzhou 310009, China

**Keywords:** low-grade glioma, brainstem gliomas (BSGs), chemoradiotherapy

## Abstract

Adult brainstem gliomas (BSGs) are rare malignant tumors of the central nervous system (CNS) that are difficult to resect completely via surgery, so chemoradiotherapy (CRT) is the primary treatment modality. However, there is substantial interpatient variability in therapeutic response to CRT among patients with BSGs. Here we present a case of a 41-year-old man who had experienced right limb weakness for 3 months. Brain MRI showed a solid-cystic mass in the brainstem, involving the medulla oblongata and the cervical spinal cord. The pathological diagnosis was pilocytic astrocytoma, CNS WHO Grade 1. Since the solid-cystic mass in the brainstem was unresectable, the patient received concurrent CRT followed by temozolomide (TMZ) maintenance chemotherapy. No significant change in tumor size was observed after concurrent CRT. Notably, 15 months after radiotherapy (19 months since the initial diagnosis), the tumor size decreased significantly. Up to the latest follow-up, the patient has maintained sustained partial remission.

**Figure 1:**
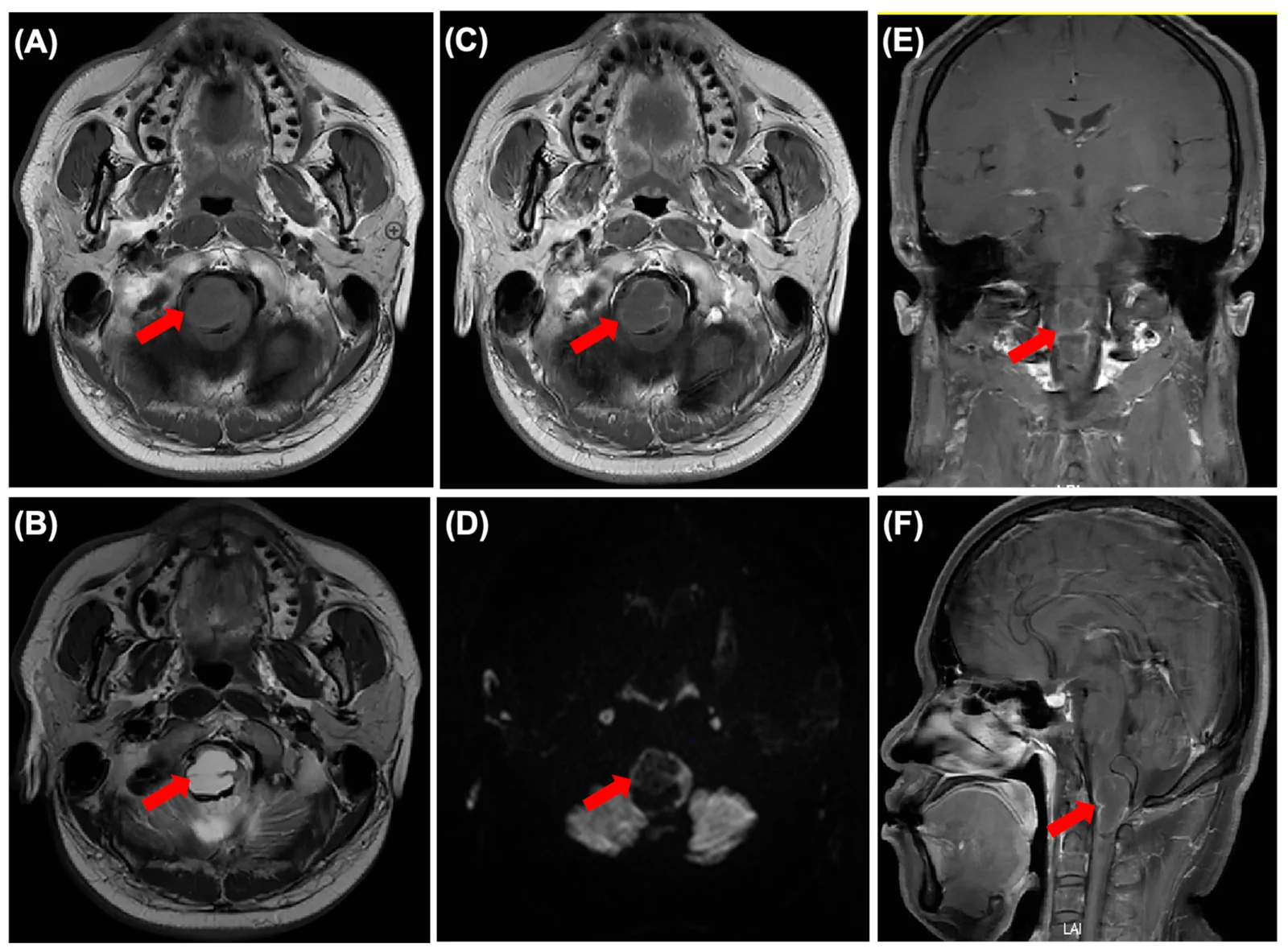
Cranial MRI showing brainstem lesions. (**A**) Axial T1WI view. (**B**) Axial T2WI view. (**C**) Enhanced T1WI view. (**D**) Axial DWI view. (**E**) Coronal enhanced T1WI view. (**F**) Sagittal enhanced T1WI view. Red arrows indicate the brainstem lesions. On 17 December 2022, a 41-year-old man presented to the Neurosurgery clinic with right limb weakness that had persisted for 3 months. Physical examination revealed normal vital signs, grade 3 muscle strength in the right upper limb, and walking instability. Contrast-enhanced cranial MRI showed a 19 mm × 22 mm × 30 mm mass with slightly low T1WI signal and slightly high T2WI signal (Figure 1A,B), and the peripheral wall of the mass showed continuous enhancement after contrast administration (Figure 1C,E,F). The DWI signal was slightly hypointense relative to the cerebellum (Figure 1D). Stereotactic biopsy was performed, and histological examination revealed slight hyperplasia of glial cells with fusiform nuclei and numerous Rosenthal fibers. No mitotic figures, necrosis, or microvessel hyperplasia was observed (Figure 2A). Immunohistochemical (IHC) staining showed that tumor cells were positive for GFAP (Figure 2B), S-100 (Figure 2C), Vimentin (Figure 2D), and Nestin, and negative for IDH1 (109) (Figure 2E), Syn, NF, NeuN, H3K27M, H3.3 G34R, CD34, and BRAF. The proliferation index of Ki-67 was 1% (Figure 2F). Molecular detection results showed IDH1 and IDH2 were wild-type, and BRAF was negative. Therefore, the HE and IHC findings were consistent with a diagnosis of pilocytic astrocytoma, CNS WHO Grade 1.

**Figure 2:**
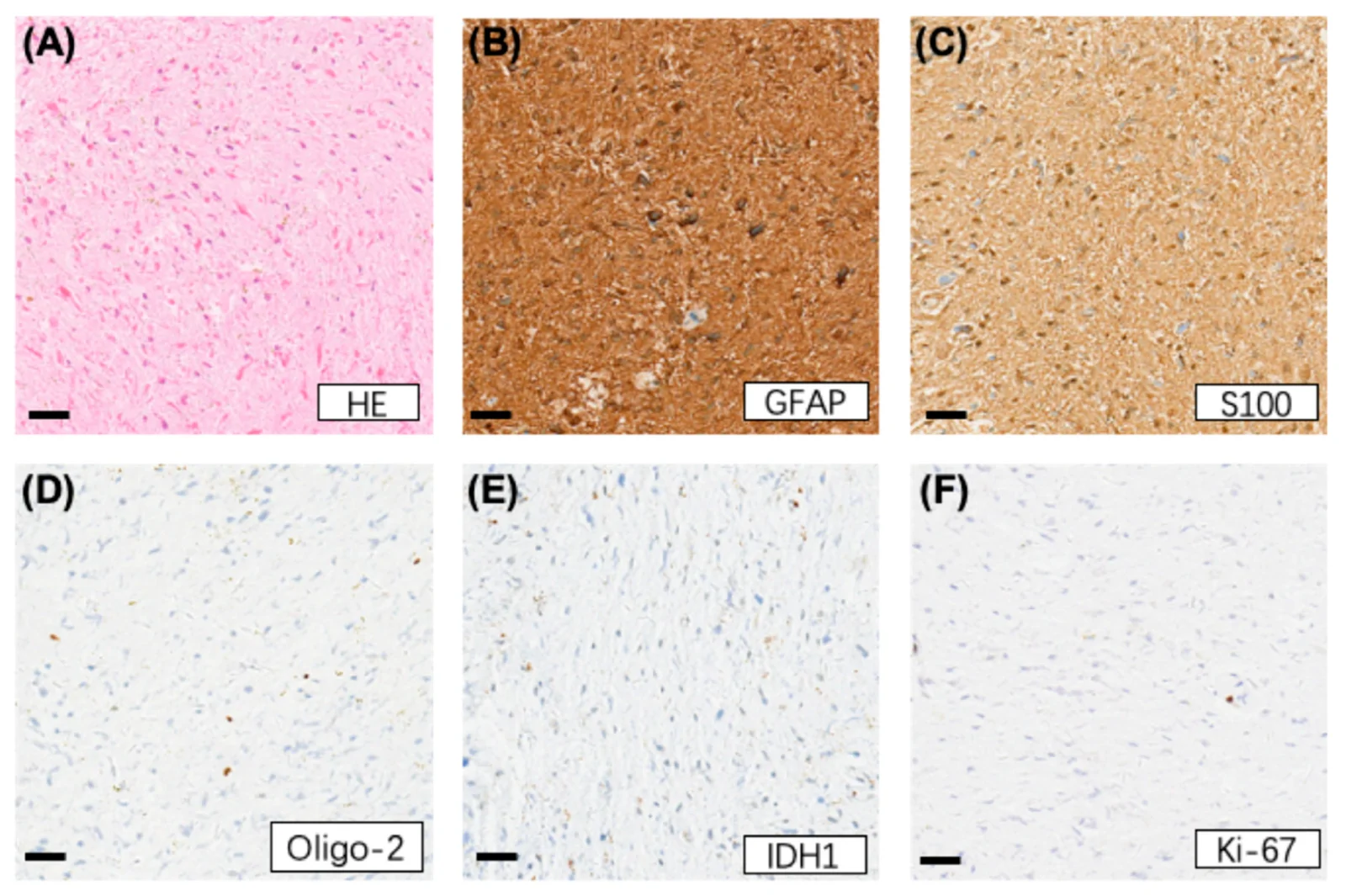
HE staining shows brainstem lesions (**A**), and IHC staining shows GFAP positivity (**B**), S-100 positivity (**C**), and Vimentin positivity (**D**) in the brainstem lesion; IDH1 staining is negative (**E**), and Ki-67 expression is low (**F**) (scale bars represent 200 µm). The patient subsequently received reduced-dose local radiotherapy at 44 Gy/22 F for brainstem lesions due to spinal cord dose constraints, and temozolomide (TMZ) chemotherapy was given concurrently. After concurrent chemoradiotherapy (CRT), contrast-enhanced cranial MRI showed the tumor still measured 19 mm × 22 mm × 30 mm with no immediate shrinkage, though the patient reported slight symptom improvement. Later, the patient took regular contrast-enhanced cranial MRI examinations every three months, and the disease remained continuously stable.

**Figure 3:**
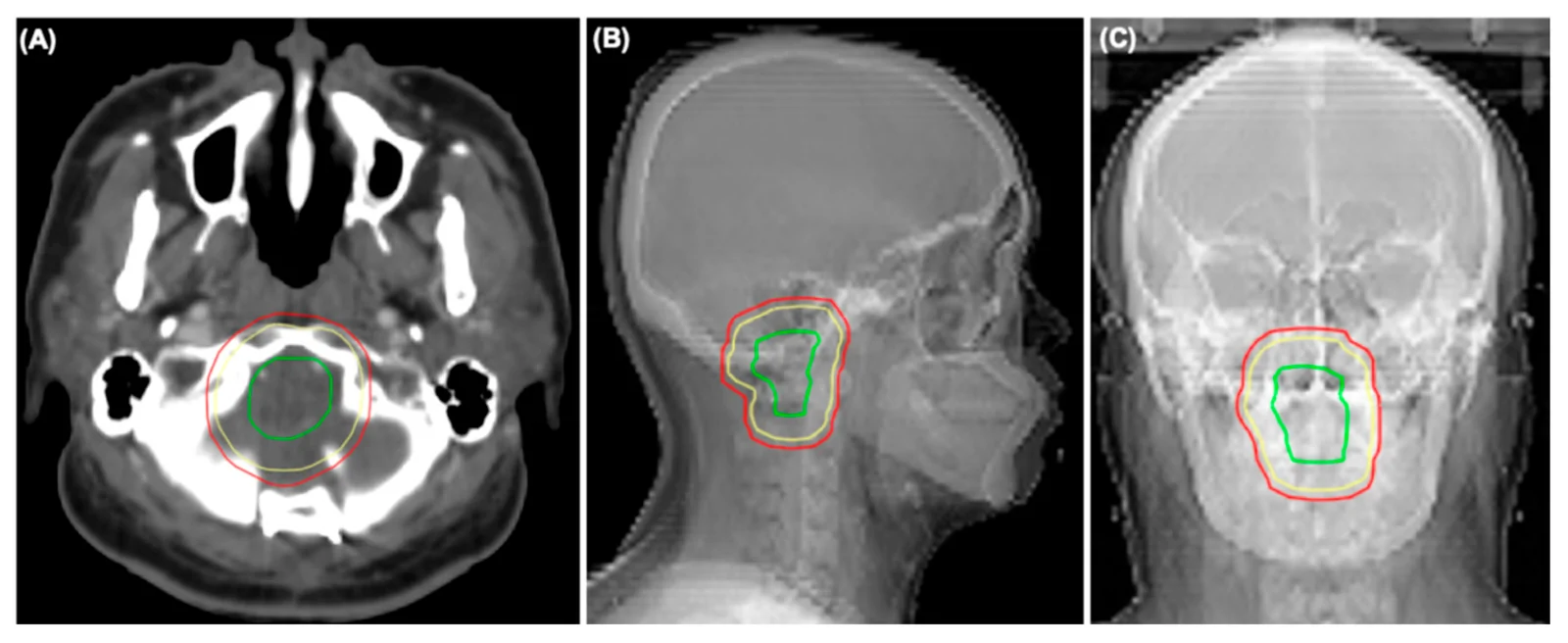
Radiotherapy fields for brainstem lesions. (**A**) Axial view. (**B**) Sagittal view. (**C**) Coronal view. Red line: gross tumor volume (GTV); yellow line: clinical target volume (CTV); and green line: planning target volume (PTV).

**Figure 4:**
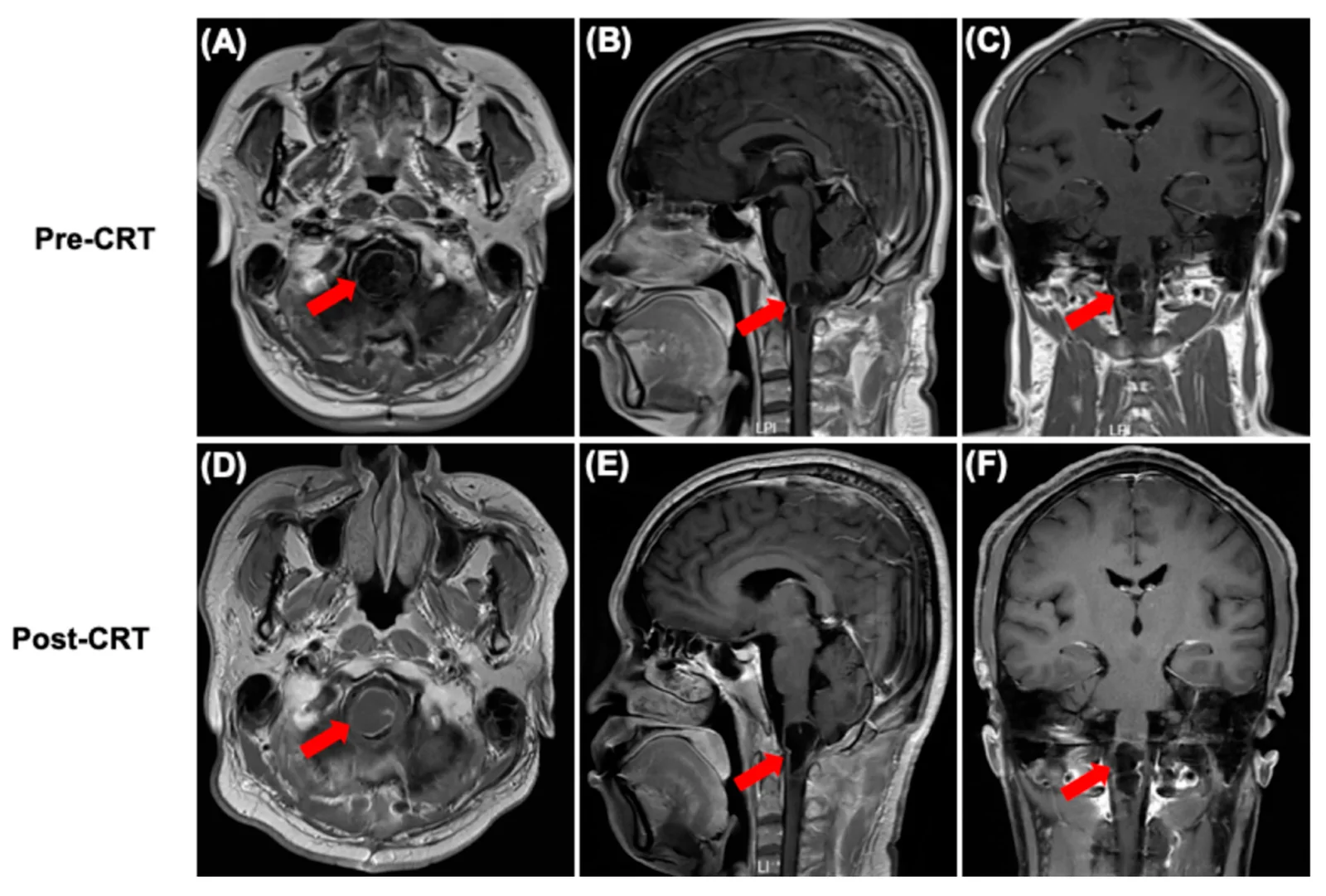
Cranial MRI showing brainstem lesions before and after CRT. (**A**–**C**) Axial, sagittal, and coronal views before CRT; (**D**–**F**) Axial, sagittal, and coronal views after CRT. The position pointed by the arrow is the site of the lesion.

**Figure 5:**
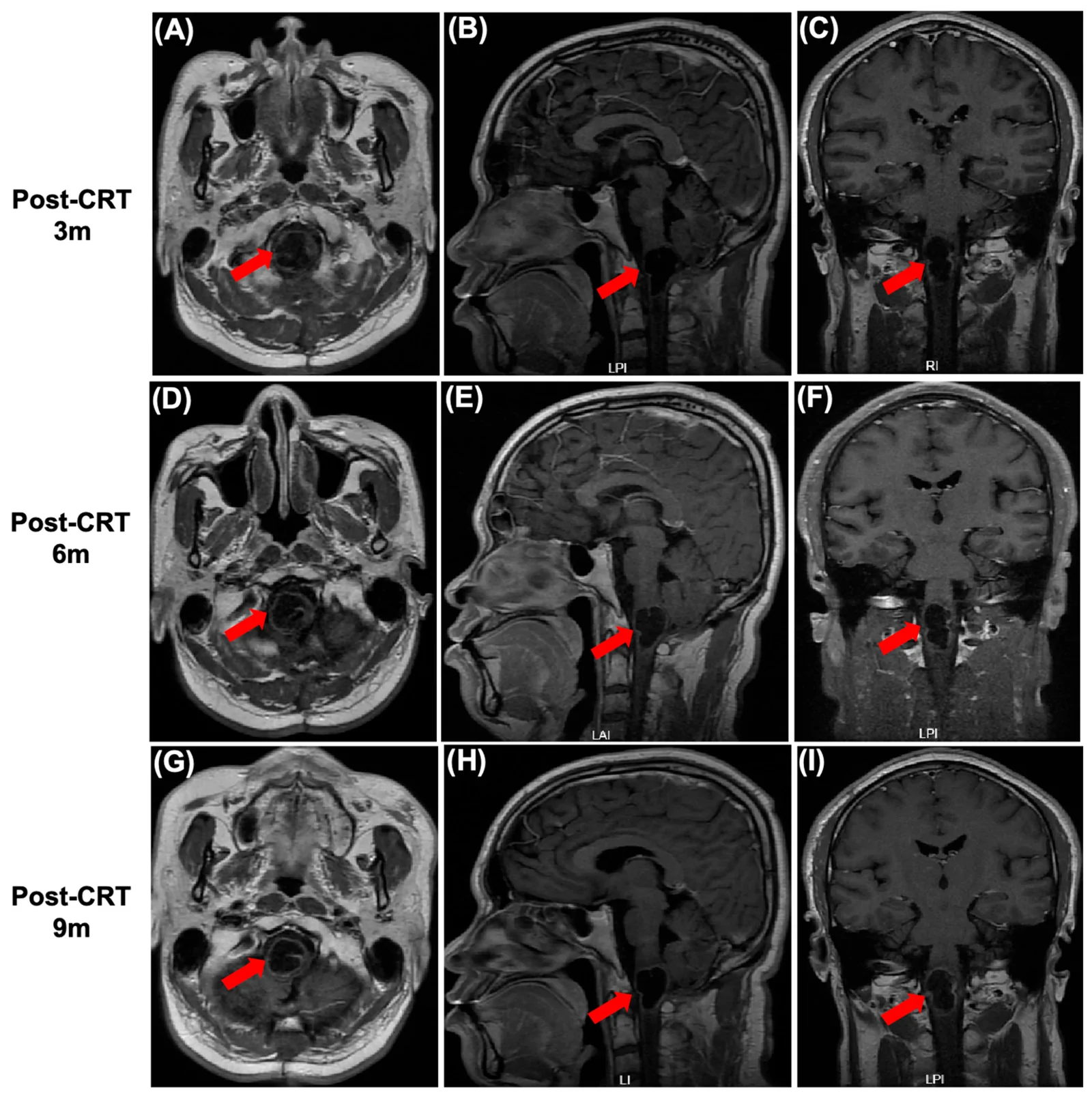
Cranial MRI images showing brainstem lesions at different time points after CRT. (**A**–**C**) Axial, sagittal, and coronal views of cranial MRI show a tumor mass measuring 20 mm × 19 mm × 33 mm 3 months after CRT; (**D**–**F**) axial, sagittal, and coronal views of cranial MRI show a tumor mass measuring 20 mm × 17 mm × 33 mm 6 months after CRT; and (**G**–**I**) axial, sagittal, and coronal views of cranial MRI show a tumor mass measuring 20 mm × 17 mm × 33 mm 9 months after CRT. The position pointed by the arrow is the site of the lesion. On 11 April 2024, he visited the clinic again 9 months after CRT, reporting that the slight worsening of right limb weakness he had experienced had persisted for nearly 2 weeks. He then received the first cycle of chemotherapy with 150 mg/m^2^ TMZ 5/23, followed by five additional cycles of 200 mg/m^2^ TMZ 5/23. After completing six cycles of TMZ chemotherapy, a significant reduction in tumor size was observed (Figure 6A–C). Subsequently, the patient underwent 12 cycles of TMZ maintenance chemotherapy. The last follow-up cranial MRI was conducted in February 2026, and the therapeutic effect was classified as continuous partial remission (PR), with a tumor mass measuring 12 mm × 11 mm × 11 mm (Figure 6D–L). His symptoms improved slightly, with only mild limping remaining, and no serious radiotherapy complications were observed during the treatment period or throughout subsequent follow-up.

**Figure 6:**
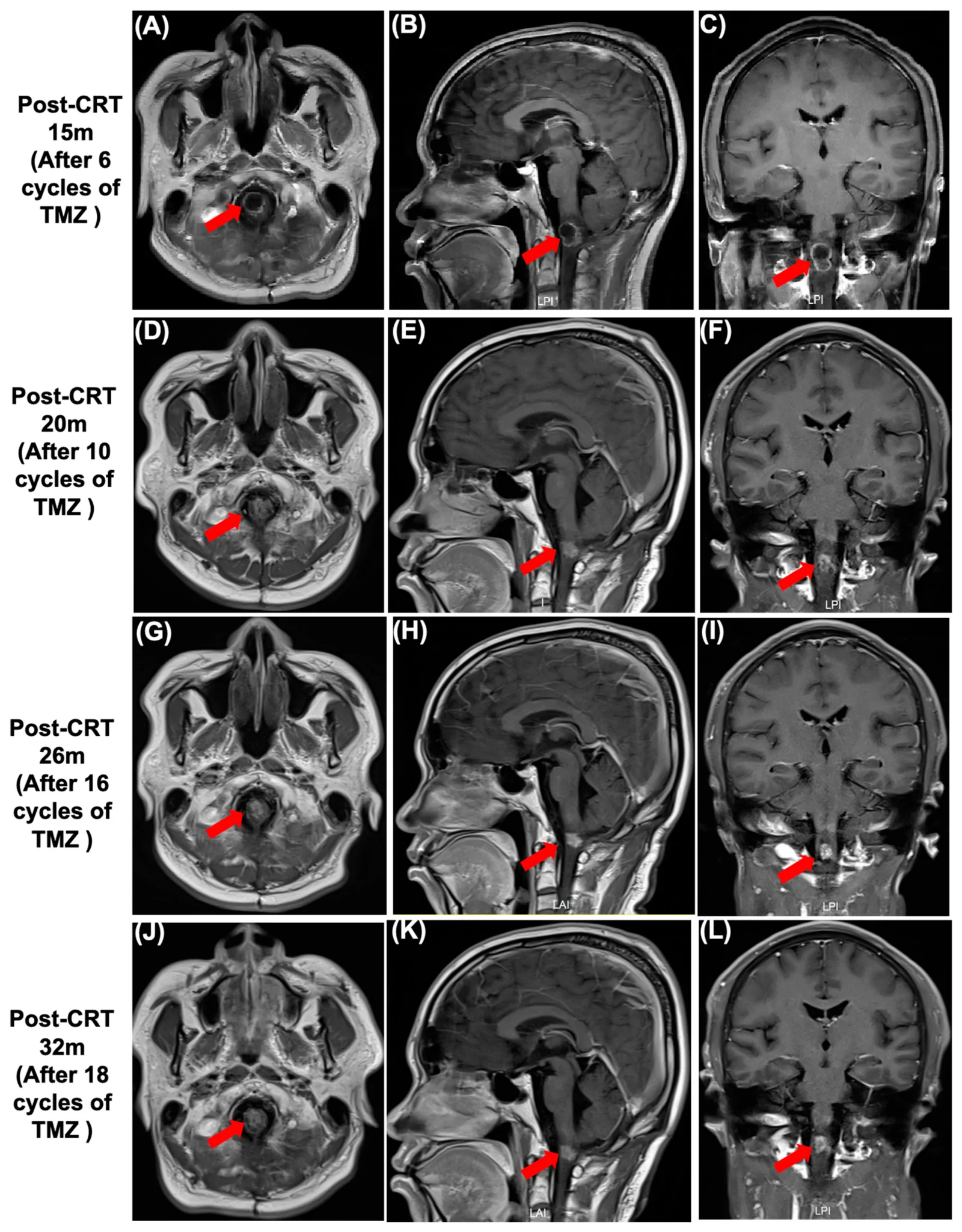
Cranial MRI images demonstrate brainstem lesions at different time points after TMZ maintenance chemotherapy. (**A**–**C**) Axial, sagittal, and coronal views of cranial MRI show a tumor mass measuring 15 mm × 13 mm × 18 mm 15 months after CRT; (**D**–**F**) axial, sagittal, and coronal views of cranial MRI show a tumor mass measuring 14 mm × 11 mm × 10 mm 20 months after CRT; (**G**–**I**) axial, sagittal, and coronal views of cranial MRI show a tumor mass measuring 12 mm × 11 mm × 10.5 mm 26 months after CRT; and (**J**–**L**) axial, sagittal, and coronal views of cranial MRI show a tumor mass measuring 12 mm × 11 mm × 11 mm 32 months after CRT. The position pointed by the arrow is the site of the lesion.

**Figure 7:**
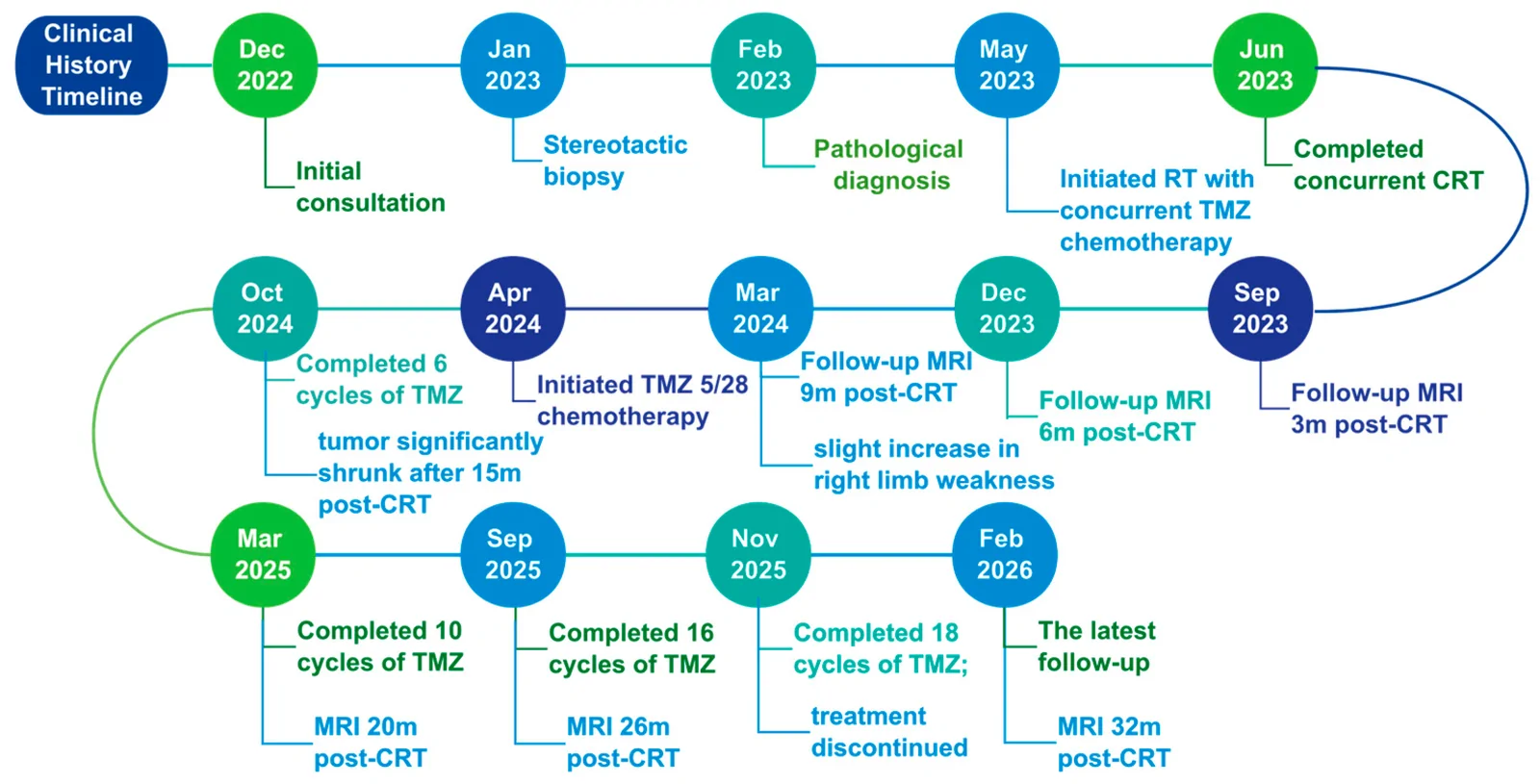
Timeline of the patient’s clinical course. Due to the low incidence, challenging location, and lack of clinical and histological data, the management of BSGs remains non-standardized. A retrospective study was conducted to analyze the characteristics, treatment patterns, and prognosis of adult patients with BSGs. The results showed that low-grade gliomas can simply be followed up or treated with radiotherapy alone [1]. Radiotherapy remains the main treatment modality for adult brainstem gliomas [2,3]. Chinese Expert Consensus on the Comprehensive Diagnosis and Treatment of Brainstem Gliomas and NCCN guideline recommend a total radiation dose of 45–54 Gy for adult low-grade gliomas [3,4]. This patient received reduced-dose local radiotherapy at 44 Gy/22 F for brainstem lesions due to the dose constraint imposed by the spinal cord’s maximum tolerated dose. A notable feature of this case is that the BSG tumor showed no reduction in size after concurrent chemoradiotherapy. However, the tumor shrank 15 months after radiotherapy, and sustained partial response (PR) was achieved throughout the follow-up period, with no obvious radiotherapy-related complications. Although tumor regression occurred during the follow-up period when the patient was administered oral TMZ, this outcome may potentially be the long-term effect of radiotherapy because the role of chemotherapy is currently unclear [5]. A 10-year retrospective single-center analysis of 81 adult patients with brainstem gliomas showed that patients can benefit from maintenance chemotherapy after radiotherapy [6]. However, this study only included seven cases of grade I gliomas, and it did not specify whether the 44 patients undergoing maintenance chemotherapy included patients with grade I brainstem tumors [6]. Although the role of chemotherapy in tumor control remains unclear, a recent case report has shown that chemotherapy-induced changes in tumor consistency can enable gross total resection of previously unresectable brainstem pilocytic astrocytoma [7]. Therefore, more aggressive treatment for brainstem LGG should be pursued due to its generally favorable prognosis. This case indicates that radiotherapy is a relatively effective and safe treatment method, even when the radiation dose cannot reach a curative level. In addition, a longer follow-up period is required to observe the therapeutic efficacy of radiotherapy. Whether maintenance chemotherapy produces survival benefits for patients with WHO grade 1 gliomas still needs to be confirmed through clinical studies.

## Data Availability

The original contributions presented in this study are included in the article. Further inquiries can be directed to the corresponding authors.

## Abbreviations

The following abbreviations are used in this manuscript:

BSGs	Adult brainstem gliomas
CNS	central nervous system
CRT	chemoradiotherapy
TMZ	Temozolomide
IHC	Immunohistochemical
RT	radiotherapy
SD	stable disease
PR	partial remission
GTV	gross tumor volume
CTV	clinical target volume
PTV	planning target volume

## References

[1] Hundsberger T., Tonder M., Hottinger A., Brügge D., Roelcke U., Putora P.M., Stupp R., Weller M. (2014). Clinical management and outcome of histologically verified adult brainstem gliomas in Switzerland: A retrospective analysis of 21 patients. J. Neurooncol..

[2] Reithmeier T., Kuzeawu A., Hentschel B., Loeffler M., Trippel M., Nikkhah G. (2014). Retrospectiveanalysis of 104 histologically proven adult brainstem gliomas: Clinical symptoms, therapeutic approaches and prognostic factors. BMC Cancer.

[3] Horbinski C., Nabors L.B., Portnow J., Baehring J., Bhatia A., Bloch O., Brem S., Butowski N., Cannon D.M., Chao S. (2023). NCCN Guidelines(R) Insights: Central Nervous System Cancers, Version 2.2022. J. Natl. Compr. Cancer Netw..

[4] Neurosurgery Oncology Group of the Chinese Medical Association (2017). Chinese Expert Consensus on the Comprehensive Diagnosis and Treatment of Brainstem Gliomas. Natl. Med. J. China.

[5] Liu Z.Y., Feng S.S., Li J., Cao H., Huang J., Fan F., Cheng L., Liu Z., Cheng Q. (2021). The survival benefits of surgical resection and adjuvant therapy for patients with brainstem glioma. Front. Oncol..

[6] Zhou J.F., Lai M.Y., Deng G.H., Zhou Z.M., Li S.Q., Zhen J.J., Li J., Hu Q.J., Cai L.B. (2022). Clinical Characteristics and Prognosis of Adult Brainstem Gliomas: A Retrospective Analysis of 81 Patients from a Single Center Over 10 Years. J. Clin. Neurosurg..

[7] Chung D.J., Arif B., Odia Y., Siomin V. (2021). Chemotherapy-induced changes in tumor consistency can allow gross total resection of previously unresectable brainstem pilocytic astrocytoma. Surg. Neurol. Int..

